# Organising care, practice and participative research: Papers from the cognitive decline partnership centre

**DOI:** 10.1111/ajag.12679

**Published:** 2019-09-08

**Authors:** Simon Biggs, Irja Haapala, Susan Kurrle

**Affiliations:** ^1^ School of Social and Political Sciences The University of Melbourne Melbourne Victoria Australia; ^2^ Brotherhood of St Laurence Melbourne Victoria Australia; ^3^ Faculty of Social Science University of Helsinki Helsinki Finland; ^4^ School of Applied Educational Science and Teacher Education University of Eastern Finland, Joensuu Campus Joensuu Finland; ^5^ Division of Rehabilitation and Aged Care Hornsby Ku‐ring‐gai Health Service Hornsby New South Wales Australia

This Special Issue of the Australasian Journal of Ageing draws on a selection of projects arising from the activities of the Cognitive Decline Partnership Centre (CDPC; https://cdpc.sydney.edu.au/), led by Professor Sue Kurrle of the University of Sydney. The CDPC is perhaps unique in that it arose from a partnership between the Australian National Health and Medical Research Council and a group of leading aged care providers, including HammondCare, Brightwater, Helping Hand plus Dementia Australia who were concerned that more research was needed to address the everyday needs, practices and experiences of those who work and live with dementia.

From the beginning of the partnership, the focus has been on generating an evidence base for improved care and support to people living with dementia and their carers. CDPC projects have focussed on four core areas: supporting the implementation of tested models of care, knowledge mobilisation of existing research, creating collaborative new research evidence and building capacity to translate research into practice. Over a five‐year period, from 2013 until the end of 2018, The Centre initiated 31 projects impacting dementia care practices across Australia. The development of the first Australian Clinical Practice Guidelines for People with Dementia was a major achievement of the program, influencing the ways care is delivered across community, residential and hospital care both nationally and internationally. A second element of the notion of partnership was an evolving and deepening relationship with the Consumers Dementia Research Network (CDRN). The CDRN, consisting of people living with dementia and carers who joined provider representatives to advise on all projects, sat on the executive and governance committees of the whole initiative. The CDRN was replaced with the Dementia Australia National Dementia Consumer Network in 2017, and members of this Network have continued to be involved. At the project level, when co‐creation worked at its best, research questions, fieldwork and the analysis of results, included discussion and debate between the members of specially created advisory groups and the research teams. Each member of an advisory group brought special expertise in order to make the research robust and relevant to the practical issues at hand.

We have selected twelve papers for this special issue that address aspects of the organisation of care, practice and research, the majority of which include collaborations between researchers, care providers, carers and people living with dementia. Flavin and Sinclair^1^ provide a commentary on the process of consumer involvement over a three‐year research initiative. Consumer and researcher perspectives are considered as part of investigating supported decision‐making in the context of dementia. They make the case for developing guidelines to support consumer involvement in dementia research.

Chen et al^2^ review the use of medication in residential aged care facilities. Residents in aged care homes, they conclude, are increasingly exposed to polypharmacy and high‐risk medications associated with medication‐related harm. This review suggests that the Australian government‐funded Residential Medication Management Review program is a useful service to identify and resolve medication‐related problems.

Biggs, Carr and Haapala^3^ explore the different perspectives people hold about the effect of dementia on daily life. Those perspectives included people living with dementia, carers, health and social work‐related professionals plus professionals from the service industries. Dementia may have social, psychological, carer role‐related, material, service‐based and disparity‐based impacts. It is argued that dementia generates its own forms of social disadvantage and exclusion that should give rise to new policy and practice priorities.

Blair, Bateman and Anderson^4^ examine how trained volunteers contributed to improvements in the emotional and physical care for older cognitively impaired patients with benefits for both staff and families. A volunteer co‐ordinator was required to carefully screen and support volunteers and foster relationships and teamwork between staff and volunteers. With clear role delineation and trained volunteers, they argue, can provide a low‐cost strategy to improve safety and care in rural hospitals.

Fitzgerald, Curry, Meierink and Cully^5^ report on a visual journey through the health‐care system as experienced by people living with dementia and their carers. The outlines of an “ideal” journey emerged, providing justification for a nationwide program of key improvements. The use of co‐created solutions through engaging consumers as co‐researchers was found to be invaluable in assisting the implementation of key themes and findings when developing dementia guidelines.

Goeman et al^6^ report on co‐design as part of a research process involving people with dementia, their care partners, aged care service experts, policymakers and academics. Lessons are drawn from a project on the key worker role in community settings. Co‐design took place across the design, conduct, implementation and outcomes, and is argued to have facilitated a robust support worker framework and a model for including the varying needs of consumers in research studies.

Haapala, Carr and Biggs^7^ report that national and local campaigning have complementary functions as change agents in addressing public health and social aspects of dementia. Policy, they suggest, should address specific priorities arising from distinctive voice perspectives, including people with dementia, carers and different professional groups, if messages are to be accepted. Interpersonal connection, advocacy and “being a normal part of society” were identified as priorities.

Harrison et al^8^ examine the differences in staffing structures between clustered and standard models of care. They argue that an increased ratio of trained personal care attendants to nursing staff does not negatively influence the quality of life. When deployed in residential aged care with increased levels of appropriate training, personal care assistants can improve the level of direct care time for the residents without negatively affecting resident outcomes.

Ratcliffe et al^9^ provide evidence on the application of a qualitative thinkaloud methodology to actively engage people with dementia and their carers in valuing particular quality‐of‐life states. People with dementia and carers used a range of decision‐making strategies, thereby facilitating assessment of the benefits of dementia care services and supports for economic evaluation from their own perspective.

Biggs and Carr^10^ examine the role of regulation in residential care for people with dementia. They found that organisations adapt using three distinctive orientations. Staff at different levels also responded, based on strategic, operational and interactional priorities in an attempt to balance the pressures of dementia care with regulatory control. They suggest that understanding how regulation is interpreted can be used to improve dementia care within a regulated environment.

Sinclair, Field, Blake and Radoslovich^11^ examine the Australian Law Reform Commission's “Decision Making Principles”^12^ for Australian aged care providers. Their paper looks at the policies of Australian aged care providers against a set of decision‐making principles based on international human rights instruments. A self‐assessment audit tool can assist aged care providers in the review of policies about health care and lifestyle decision‐making.

Kurrle et al^13^ explore the implementation of a model of care for hospitalised older patients with cognitive impairment, called the CHOPS program. In six New South Wales hospitals, increases in cognitive screening, risk identification, assessment and the management of cognitive impairment, plus communication with carers, were found. Improvements in staff knowledge and confidence, plus environmental changes, occurred.

Overall, these papers provide a useful snapshot of key projects in the work of the Cognitive Decline Partnership Centre. We hope that they encourage you to visit the CDPC website (https://cdpc.sydney.edu.au/), where a variety of full reports, guidelines and other materials can be found.
